# Supplementary material for: Patterns of Intron Gain and Loss in Fungi
Source: PLoS Biol. 2004 Nov 30;2(12):e422. doi: 10.1371/journal.pbio.0020422 (PMC532390; doi:10.1371/journal.pbio.0020422)
Supplement: Table S1 — Also available at http://genes.mit.edu/NielsenEtAl/. (4.3 MB ZIP). [file pbio.0020422.st001.zip › NielsenEtAl/html/825.html]

AN9438.1.NCU07993.1.MG01758.1.FG05090.1


```
 CLUSTAL W (1.82) Multiple Sequence Alignments - Introns Inserted


Sequence 1: MG01758.1	370 aa
Sequence 2: FG05090.1	375 aa
Sequence 3: NCU07993.1	480 aa
Sequence 4: AN9438.1	382 aa
Alignment Length: 507 aa
Number Identitical Residues: 131 aa
Alignment Score (without introns) 6244


MG01758.1 	---------------------------------------------~----MSAPP-SIPN
NCU07993.1	MERAVENDGKCPVRHWRRGPSEAIHPHGTYLTLTIQVGKRPLDTQ2TAYLMSGSAPSIPN
FG05090.1 	---------------------------------------------~----MSGP--EIPN
AN9438.1  	---------------------------------------------~----MSAP--QIPN
          	                                                  **..  .***

MG01758.1 	LFS-LRGARGGGGFRQRRG------GGG---GVAQSAAGHDATVQGTDTDAAVSRSSAVG
NCU07993.1	LLTLRGRIGGGGVTRGRYRGVIHRGGRGHGPGAPSASAAHDATIQGTDTDAAVSRLSAVQ
FG05090.1 	LLSSLRSARGG---RGRGR------GRG---GHASSSATHDSTIQGTDTDASVSRLSAVD
AN9438.1  	LNTLRRGGGRG---RFRAR------GGP---DSSSSSGNKDRVVQGTDNDASVSRLSAVE
          	* :       *   * *        *     . ..::. :* .:****.**:*** *** 

MG01758.1 	LGYLDDPFAQYFVQNAVGPSSRRLPIINRG1TYTRTAGLDVLVDRFLGVADGSQRQQQQG
NCU07993.1	IGYIDDPYAELFAQSGPG-AARRLPIINRG~TYARTTAIDKLVDKFLDDT-------ESS
FG05090.1 	LGYLDDPYAQYFVRSSAGPAARRLPIINRG1TYARTISLDTLIESFLSAD------QSTG
AN9438.1  	LGYLEDPFARALTP--MGQEMRRLPIINRG1TYVRTTAIDQLVASFLGLKAD----SDPT
          	:**::**:*. :.    *   ********* **.** .:* *:  **.   .    ..  

MG01758.1 	QEPLQRQIISLGAGTDTRSLRLLNRHG---AAGIVYHEIDFPLICAKKLNIVKATPALRN
NCU07993.1	PE--GRQIVSLGAGTDTRSLRLFSPSAPTPRKRVIYHEIDFPAMCEKKQRIVCSAPQLRS
FG05090.1 	PDSTPKQIVSLGAGTDTRPFRLFASKA---RPGLVYHELDFEVVTSKKLRTVQAVPLLRN
AN9438.1  	WKLKKKQIISLGAGSDTRVFRLLSLRP---ALDIIYHEIDFAVNNTAKIKAIQGTPLLQR
          	 .   :**:*****:*** :**:          ::***:**      * . : ..* *: 

MG01758.1 	LLTDPQVEDDG-------SWQC----STPNNGRFWCHGLDLRKLTS--------------
NCU07993.1	ILSDPDSVEELSQHGGGNSWHSKAVAEKHKGSELWVHGLDLRAIAASQQPQQPLPPGVPI
FG05090.1 	ILTNVTQLTEH-------SWS-----SKPSGGEYYCHGQDLRNFSLSKASK---------
AN9438.1  	VLGQSQVSISNE-------------GDELHSPAYHIHAVDLRTLAQKGEGD---------
          	:* :     . .             ..   .     *. *** :: .   .         

MG01758.1 	------------------------QQQPD--ASVDIKGLRTDIPTLIISECCLCYLQVSE
NCU07993.1	GSRGLHASPFTPGSTTQHEEQTEETSLPQQREPLTLTSLNPNLPTLIISECCLCYLPPST
FG05090.1 	-------------------EYHNTQDAPQEKPEITLPGLRTDIPTLLLSECCLCYLNGTE
AN9438.1  	------------------------KSTGQDPGRRLQDFVDTTLPTLLLSECCLIYLSPND
          	                         .  :.        : . :***::***** **  . 

MG01758.1 	AKSVTKWFSDRIPS-------LGIVIYEPTKPDDPFGKTMISNLAARSISMPTLSIYRQP
NCU07993.1	ASSIVSFFTTTIQSS------LSIVIYEPIKPDDAFGKMMVSNLAAREIRMPTLEVYKEA
FG05090.1 	ASDVLNFFTSRIPN-------LATIIYEPIRPDDAFGKMMVSNLAARRIEMPTLKMYPTP
AN9438.1  	AAGVVRYFTHTLFPASQETETLALVLYEPIRPDDAFGRTMVANLATRGIQLQTLHQYASL
          	* .:  :*:  :  ::..:.:*. ::*** :***.**: *::***:* * : **  *   

MG01758.1 	ADQDTRLRDAGFEK--------ARHMTVKDIWSQWVAPEEKERVDRLEGLDEVEEWDLLA
NCU07993.1	EDQERRLREAGFSGGEGKGIGGARSKTIEQIWEEWTSQEEKERVDALEGLDEVEEWKLLA
FG05090.1 	EDQRTRLRTAGFET--------VHHMTIEDIWQNWVSPEEKQRVDYLEGLDEVEEWKLLA
AN9438.1  	GAQRQRLREHGFNGG-------QAAADVDFLWERWVAEEEKERVAALEMLDEVEEWKLLA
          	  *  ***  **. .            :. :*..*.: ***:**  ** *******.***

MG01758.1 	GHYVVAWAWR--GLGFEPWENLR------
NCU07993.1	GHYIVVWGWR--GVGVD----LEI-----
FG05090.1 	AHYIVVWASR--GSGFGNWGGVGVGA---
AN9438.1  	QHYCVAWGWRDGSTRFTGWGDLDGQSAEE
          	 ** *.*. *...  .    .:   ::..
```
